# Clinical application of targeted next-generation sequencing in pneumonia diagnosis among cancer patients

**DOI:** 10.3389/fcimb.2025.1497198

**Published:** 2025-02-18

**Authors:** Ke Yang, Jiuzhou Zhao, Tingjie Wang, Zhizhong Wang, Rui Sun, Dejian Gu, Hao Liu, Weizhen Wang, Cuiyun Zhang, Chengzhi Zhao, Yongjun Guo, Jie Ma, Bing Wei

**Affiliations:** ^1^ Department of Molecular Pathology, The Affiliated Cancer Hospital of Zhengzhou University & Henan Cancer Hospital, Zhengzhou, China; ^2^ Medical Department, Geneplus-Beijing Co., Ltd., Beijing, China

**Keywords:** targeted next-generation sequencing, pathogen, cancer patients, CMT, pneumonia

## Abstract

**Background:**

Cancer patients are highly susceptible to infections due to their immunocompromised state from both the malignancy and intensive treatments. Accurate and timely identification of causative pathogens is crucial for effective management and treatment. Targeted next-generation sequencing (tNGS) has become an important tool in clinical infectious disease diagnosis because of its broad microbial detection range and acceptable cost. However, there is currently a lack of systematic research to evaluate the diagnostic value of this method in cancer patients.

**Methods:**

To evaluate the diagnostic value of tNGS for cancer patients with pneumonia, a retrospective analysis was conducted on 148 patients with suspected pneumonia who were treated at the Henan Cancer Hospital. The tNGS results were compared with conventional microbiological tests (CMT) and clinical diagnoses based on symptoms and imaging studies to assess the diagnostic performance of tNGS in cancer patients with pneumonia.

**Results:**

Among these 148 patients, 130 were ultimately diagnosed with pneumonia. tNGS demonstrated significantly higher sensitivity (84.62% vs. 56.92%) and diagnostic accuracy (85.81% vs. 62.16%) compared to the CMT method. The tNGS method identified more pathogens than CMT method (87.50% vs 57.14%), regardless of whether they were bacteria, fungi, or viruses, primarily due to its broader pathogen detection range and higher sensitivity compared to the CMT method. tNGS had significantly higher diagnostic accuracy for *Pneumocystis jirovecii* and *Legionella pneumophil*a than the CMT method, but for most pathogens, tNGS showed higher sensitivity but with a correspondingly lower specificity compared to CMT.

**Conclusion:**

tNGS demonstrates higher sensitivity and a broader pathogen detection spectrum compared to CMT, making it a valuable diagnostic tool for managing pneumonia in cancer patients.

## 1 Introduction

Cancer patients are highly susceptible to infections due to their immunocompromised state, which results from both the malignancy and the intensive treatments they undergo. Accurate and timely identification of the causative pathogens is crucial for the effective management and treatment of these infections. Traditional diagnostic methods, such as blood cultures, often have significant limitations, including low sensitivity (Babady, 2016), limited scope of pathogen detection (Hill et al., 2024; Rodino and Simner, 2024) and prolonged turnaround times. These limitations can delay appropriate treatment and negatively impact patient outcomes (Deng et al., 2022; Gu et al., 2023).

Multiplex polymerase chain reaction(PCR) is a commonly used molecular detection technique in clinical settings. Its ultra-high sensitivity provides support for precise clinical diagnosis (Hou et al., 2020; Zhang et al., 2022). However, considering its limited target coverage, the clinical application is somewhat restricted. Next-generation sequencing (NGS) has emerged as a powerful tool in the detection of infectious agents. Metagenomic NGS(mNGS) has garnered significant clinical acclaim in recent years, offering the unprecedented ability to detect all nucleic acids in a sample without prior assumptions (Chiu and Miller, 2019). Numerous studies have demonstrated that mNGS far surpasses traditional diagnostic methods in terms of diagnostic value for infections (Liang et al., 2022; Serpa et al., 2022; Fourgeaud et al., 2024). However, the cost of over 500 USD per test has led to its recommendation primarily for the diagnosis of critically ill patients.

As the new favorite in clinical settings following metagenomic NGS (mNGS), the clinical performance of tNGS has been demonstrated through several studies. In the diagnosis of respiratory tract infections, the diagnostic performance of tNGS is comparable to that of mNGS, but the cost is less than half (Wei et al., 2024; Zhang et al., 2024). In patients with lower respiratory tract infections and pneumonia, tNGS has shown better sensitivity than traditional methods and has diagnostic value comparable to that of mNGS (Gaston et al., 2022; Li et al., 2022; Zhang et al., 2023; Zhang et al., 2024), but its specific application in the context of cancer patients warrants further investigation. This population presents unique challenges, such as a higher prevalence of opportunistic infections (Deng et al., 2023) and the presence of non-pathogenic microorganisms that may complicate the interpretation of sequencing results.

In this study, we aim to evaluate the diagnostic performance of tNGS in cancer patients with pneumonia. We compared tNGS results with those obtained from conventional microbiological tests, and a clinical diagnosis to assess its sensitivity, specificity, and accuracy. Furthermore, we have analyzed the pathogen spectrum detected by tNGS in this patient population. Through this comprehensive analysis, we hope to elucidate the potential of tNGS as a reliable and efficient diagnostic tool for managing infections in cancer patients.

## 2 Methods

### 2.1 Sample enrollment and microbiology testing

**Figure 1:**
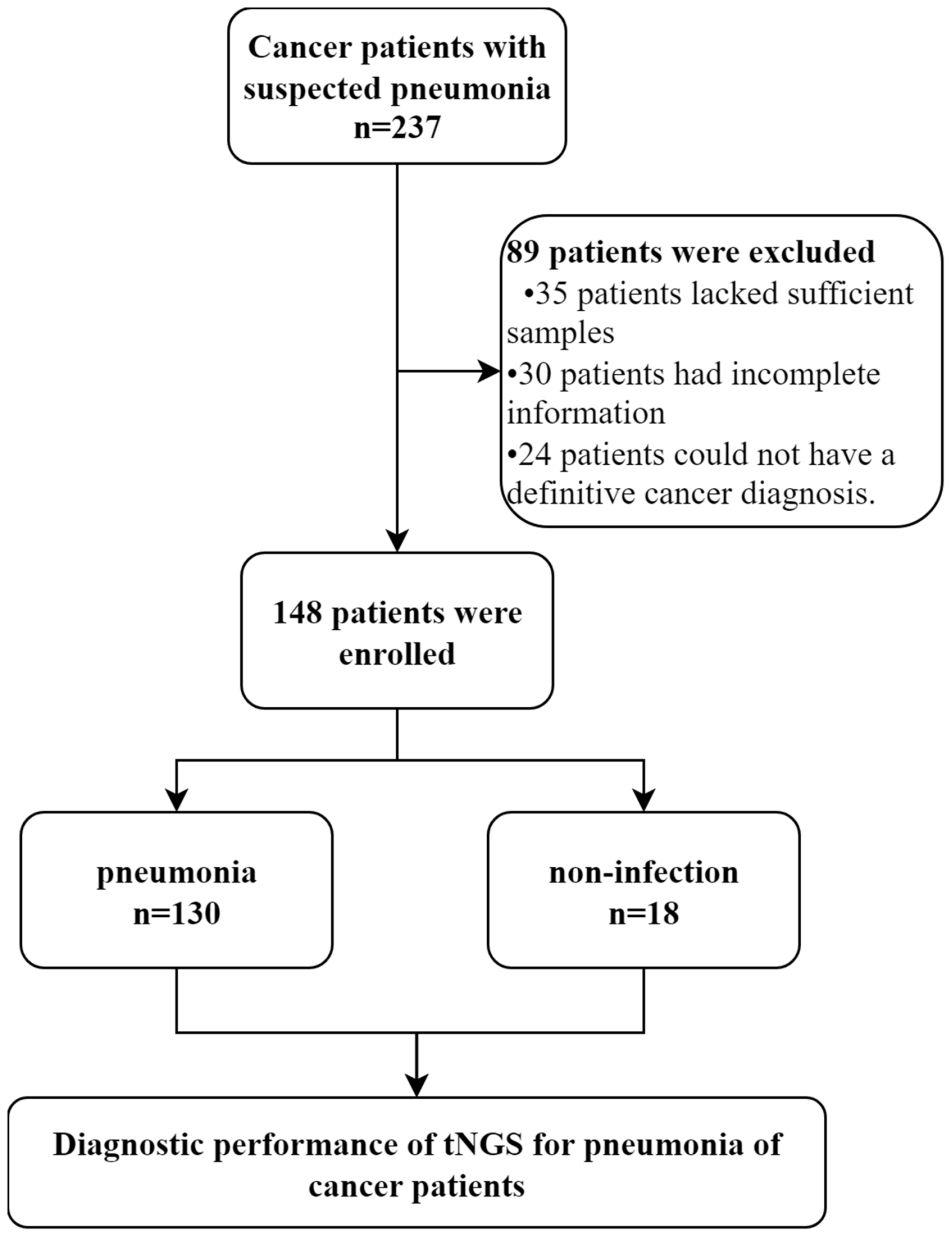
Flowchart of samples analyzed by tNGS. We conducted a retrospective analysis of eligible patients and ultimately included 148 participants. Patients were divided into pneumonia and non-infection groups based on their final clinical diagnoses to explore the diagnostic performance of tNGS.

To evaluate the diagnostic performance of tNGS in pneumonia of cancer patients, a retrospective study was conducted, enrolling 237 cancer patients with suspected pneumonia who had undergone tNGS testing at the Henan Cancer Hospital from April 2022 to April 2024(**Figure 1**). Clinical information and biochemical indicators were collected through the hospital’s electronic medical record system, and ultimately, 148 patients with complete clinical information and diagnostic outcomes were included in the analysis. Patients were eligible for enrolment if they (1) were at least 18 years of age; (2) were undergoing cancer treatment; (3) had suspected pneumonia. Patients with suspected pneumonia met both of the following criteria: (1) at least one compatible symptom, such as new-onset fever, cough, or dyspnea; (2) new-onset radiological findings on chest images.

This study received approval from the Ethics Committees to ensure compliance with ethical guidelines. Patients’ identification remained anonymous throughout the study, and informed consent was waived due to the retrospective and observational nature of the research. All samples were aliquoted into 1ml portions and stored at -80°C after clinical collection.

### 2.2 Conventional microbiological tests

All CMTs were performed on every sample, except for viral PCRs, which were performed at the clinician’s discretion. A total of 148 patients underwent BALF sampling, from which a portion was aliquoted for testing by tNGS. Additionally, this BALF was subjected to CMTs alongside other relevant samples, such as blood and urine. The CMTs encompassed a range of diagnostic methods including culture, antigen detection, multiplex PCR, and Xpert PCR assays.

### 2.3 tNGS workflow for BALF

tNGS was performed once the sample was obtained. As previous reported (Wei et al., 2024; Zhang et al., 2024), BALF samples with high viscosity were diluted 1:1 with 0.1 M dithiothreitol before nucleic acid extraction. A volume of 400μL of BALF, lysis buffer, protease K mixture, binding buffer, and 1.2 g glass bead were agitated vigorously at 4500 rpm for a total of 130 s by FastPrep-24™ 5G Instrument (MP Biomedical, CA, USA). Nucleic acid extraction was performed using the VAMNE Magnetic Pathogen DNA/RNA kit (Vazyme, Nanjing, China). Then, the RNA was reverse-transcribed into cDNA using Hieff NGS^®^ ds-cDNA Synthesis Kit (Yeasen, Shanghai, China). Nucleic acids were quantified using a Qubit 3.0 fluorometer. A549 human cells (GenePlus, Suzhou, China) were used as negative controls (NTC) to detect contamination, and A549 human cells spiked with *Staphylococcus aureus* (BeNa Culture Collection, Beijing, China) were used as positive controls (PTC). Following extraction, cDNA synthesis and library preparation were performed with the HieffNGS^®^C37P4 One PotcDNA&gDNA Library Prep Kit (Yeasen, Shanghai, China). Then, the cDNA was taken through library enrichment with NadPrep^®^ NanoBlockers (Nanodigmbio, Nanjing, China) reagents to generate the product for targeted sequencing. Eight uniquely barcoded libraries were pooled to hybridize and capture by specific biotinylated probes for 4 hours using Geneplus design probes. Products were quantified with a Qubit 3.0 instrument using DNA HS Assay Kit (Vazyme, Nanjing, Jiangsu, China). Products were stored at −20°C until Sequencing. After library construction, sequencing was performed on the Gene+seq 100 platform(GenePlus, Suzhou, China). The sequencing read length was set to 100 base pairs (bp), with a preset data of 5 million reads. Clean reads were obtained by removing sequencing adapters, low-quality reads, or reads below 35 bp using fastp (version 0.23.1). The remaining reads were aligned to the human reference (hg38) using Burrow-Wheeler Aligner (version 0.7.12-r1039) and human reads were filtered. The filtered reads were compared with the self-built pathogenic microorganism database, and the retained results were annotated. Microbial reads within the target range were normalized to reads per million (RPM), and only microorganisms above a predefined threshold were initial reported in this study. The threshold was set at RPM ≥6 for common pathogens (excluding mycobacteria) and ≥0.5 for fungi and mycobacteria. A manual review is conducted. The common colonizing microorganisms within the respiratory tract target range was reported separately from other microorganisms in different sections to avoid interfering with clinical judgment (Li et al., 2023; Zhang et al., 2024).

### 2.4 Clinical diagnosis as the reference standard

Two physicians with extensive clinical experience, each having worked in the respiratory department for over 5 years, independently reviewed all inpatient medical information and microbiological test results of the patients, including tNGS. Initially, they determined whether the patients had infectious diseases. Subsequently, they identified the pathogenic microorganisms based on clinical manifestations, laboratory tests, imaging examinations, microbiological test results (including CMT and tNGS), and treatment responses. Discrepancies between the two reviewers were first resolved through in-depth discussions; if consensus could not be reached, another senior reviewer was consulted.

### 2.5 Statistical methods

For continuous variables, report as the median and interquartile range (IQR). Categorical variables are represented by frequency and percentage. Inter-group comparisons are made using the unpaired t-test or the Mann-Whitney U test. For comparisons between groups of categorical variables, the chi-square test is used. In evaluating diagnostic performance, sample consistency is judged by referring to previous studies (Blauwkamp et al., 2019); partial pathogen consistency is considered as consistent. In the calculation of diagnostic performance, sensitivity, specificity, and accuracy are computed using the standard proportion formula, and the 95% confidence intervals for these proportions are determined using the Wilson method. To compare the differences in diagnostic performance and analyze diagnostic accuracy between two groups, a t-test can be employed.

All figures were drawn using GraphPad Prism version 9.5.0 (GraphPad Software LLC., San Diego, CA, USA). All analyses were performed with SPSS version 26.0 (SPSS Inc., Chicago, Illinois, USA) (Blauwkamp et al., 2019). A p-value less than 0.05 was considered statistically significant.

## 3 Results

### 3.1 Patient characteristics

A total of 237 patients with suspected pneumonia were considered for inclusion. Of these, 89 patients were excluded, some because they were ultimately diagnosed as non-cancer patients, and others due to insufficient data for further analysis. One hundred and forty-eight patients met the criteria and were included in the final analysis. This group comprised 60.14% male patients. The patients were primarily composed of three types of cancer patients, with lung cancer accounting for 69.59%, hematological malignancies accounting for 18.24%, and gastrointestinal cancer accounting for 8.78%. Additionally, there were 5 patients mainly with ovarian, bladder, and breast cancer (**Table 1**). When comparing pneumonia and non-infection patients, no significant differences in characteristics were observed between the two groups. However, the proportion of hematological malignancies was higher in the pneumonia patient group.

**Table 1:** Baseline characteristics of the 148 patients enrolled.

Characteristic	Number (n = 148)	Pneumonia (n=130)	non-infection (n=18)	p value
**Median age, years**	61(16-83)	60(16-81)	66(41-83)	0.122
**Gender, n(%)**				0.928
Male	89 (60.14)	78(60.00)	11(61.11)	
Female	59 (39.86)	52(40.00)	7(38.89)	
Laboratory findings				
WBC (10^9^/L), median (IQR)	7.41 (5.75, 10.56)	7.49(5.73, 10.76)	6.41(5.58, 9.25)	0.609
NEUT%, median (IQR)	73.44 (63.21, 82.63)	73.44(63.35, 83.45)	67.75(61.20, 80.13)	0.371
LYM%, median (IQR)	17.42 (8.97, 27.82)	16.52(8.65, 27.65)	19.95(10.55, 28.925)	0.446
CRP(nmol/L),median (IQR)	87.88(49.31, 158.13)	89.32(51.22, 153.33)	86.13(48.34, 153.27)	0.433
PCT(ng/mL),median (IQR)	1.41(0.48,5.32)	1.57(0.46, 5.77)	1.26(0.37, 5.21)	0.189
Cancer types, n(%)				
lung cancer	103 (69.59)	98(75.38)	15(83.33)	0.238
hematological malignancies	27 (18.24)	27(20.77)	0(0)	0.03
gastrointestinal cancer	13 (8.78)	11(8.46)	2(11.11)	0.709
other cancers	5 (3.38)	4(3.08)	1(5.56)	0.585

IQR, interquartile range; WBC, White blood cell; PCT, procaicitonin; CRP, C-reactive protein; LYM, Lymphocyte; NEUT, Neutrophil. Other cancer types included two patients with ovarian cancer, two with breast cancer, and one with bladder cancer. Bold formatting is used to denote the primary groupings.

### 3.2 Clinical diagnostic performance of tNGS and CMT

In the clinical diagnosis, 130 patients were confirmed to have infections, while 18 patients were non-infectious. tNGS identified 110 infected patients and found no microorganisms in 17/18 non-infectious patients, ultimately showing a sensitivity of 84.62% and a specificity of 94.44%, with a diagnostic accuracy of 85.81% (**Table 2**). The sensitivity of CMT in diagnosing was 56.92%, with a specificity of 100%, and a diagnostic accuracy of 62.16%. The diagnostic accuracy of tNGS was significantly higher than that of the CMT method. This was mainly because tNGS had significantly higher sensitivity for fungal and bacterial infections compared to the CMT method (p<0.0001). However, due to the excessive sensitivity of tNGS, there was no significant difference in accuracy between tNGS and CMT for bacterial and fungal infections. In terms of viral infections, PCR, as the diagnostic standard, had a higher diagnostic accuracy than the tNGS method. Nevertheless, the broader coverage of viruses by tNGS provided better sensitivity. Therefore, tNGS was a more sensitive method than CMT overall.

**Table 2:** Diagnostic performance of tNGS in 148 patients.

	Methods	Sensitivity(95% CI)	Specificity(95%CI)	PPV(95% CI)	NPV(95%CI)	Accuracy(95%CI)	p-value
**All samples**	**CMT**	56.92% (48.33%-65.12%)	100.00% (82.41%-100.00%)	100.00% (95.07%-100.00%)	24.32% (15.98%-35.21%)	62.16% (54.13%-69.57%)	<0.0001
	**tNGS**	84.62% (77.43%-89.81%)	94.44% (74.24%-99.01%)	99.10% (95.07%-99.84%)	45.95% (31.04%-61.62%)	85.81% (79.28%-90.53%)	
**Fungal pneumonia**	**CMT**	52.63% (37.26%-67.52%)	99.09% (95.03%-99.84%)	95.24% (77.33%-99.15%)	85.83% (78.71%-90.84%)	87.16% (80.82%-91.63%)	0.86
	**tNGS**	81.58% (66.58%-90.78%)	88.18% (80.82%-92.96%)	70.45% (55.78%-81.84%)	93.27% (86.75%-96.70%)	86.49% (80.05%-91.08%)	
**Viral pneumonia**	**CMT**	76.09% (62.06%-86.09%)	100.00% (96.37%-100.00%)	100.00% (90.11%-100.00%)	90.27% (83.41%-94.48%)	92.57% (87.18%-95.80%)	0.0005
	**tNGS**	89.13% (76.96%-95.27%)	78.43% (69.50%-85.30%)	65.08% (52.75%-75.67%)	94.12% (86.96%-97.46%)	81.76% (74.76%-87.15%)	
**Bacterial pneumonia**	**CMT**	57.53% (46.10%-68.22%)	89.33% (80.34%-94.50%)	84.00% (71.49%-91.66%)	68.37% (58.62%-76.73%)	73.65% (66.02%-80.08%)	0.17
	**tNGS**	84.93% (75.00%-91.37%)	76.00% (65.22%-84.25%)	77.50% (67.21%-85.27%)	83.82% (73.31%-90.72%)	80.41% (73.28%-86.00%)	

tNGS, targeted Next-generation sequencing; CMT, conventional microbiological tests; PPV, positive predictive value; NPV, negative predictive value; NA, not available. The difference of diagnostic accuracy based on the t-test of tNGS and CMT was marked as p-value. Bold formatting is applied to emphasize the subgroups and detection methods.

### 3.3 Pathogens identification performance of tNGS

**Figure 2:**
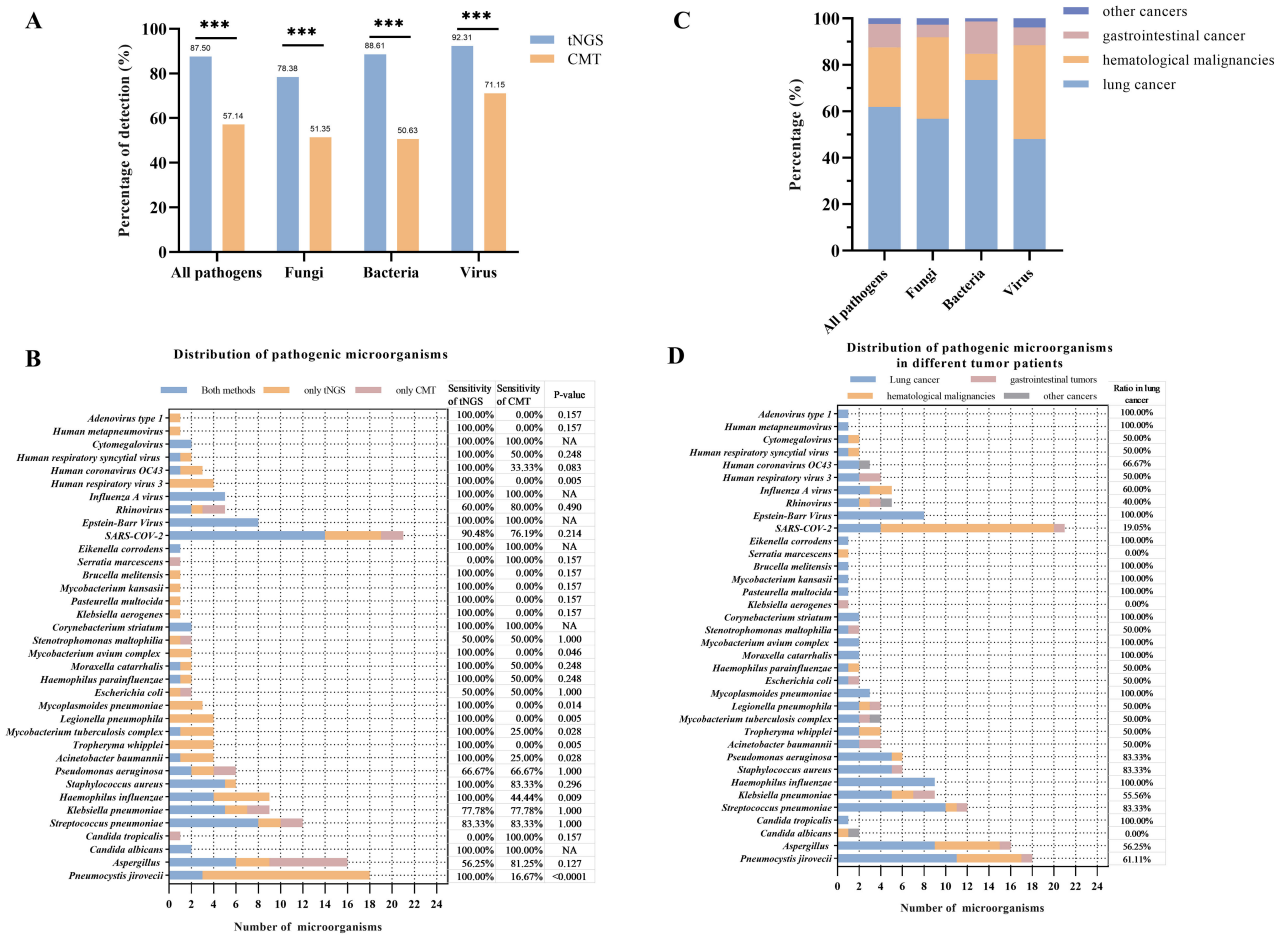
Comparison between tNGS and CMT in pathogens detection. **(A)** Percentage of pathogens detection in difference methods. **(B)** Distribution of pathogens and comparison between tNGS and CMT. The sensitivity of tNGS or CMT was calculated as follows: sensitivity = pathogens detected by tNGS or CMT/Total. The difference of sensitivity based on the t-test of tNGS and CMT was marked as p-value. **(C)** Percentage of pathogens founding in different cancer patients. **(D)** Distribution of pathogens in different cancer patients. *p<0.05, **p<0.01, ***p<0.001..

The study further explored the diagnostic accuracy of the targeted Next-Generation Sequencing (tNGS) method for various pathogens. Ultimately, 168 pathogens were identified in these patients. Of these, tNGS identified 147(87.50%), while CMT identified 96(57.14%). The tNGS method detected a significantly higher number of pathogens compared to CMT (p<0.0001), including bacteria, fungi, and viruses (**Figure 2A**). A further comparison of the pathogen distribution profiles revealed that tNGS had higher sensitivity for detecting *Pneumocystis jirovecii* among fungi (**Figure 2B**). For viruses, tNGS was able to identify additional RNA viruses not covered by multiplex PCR, such as *Human respiratory viruses*, *Human metapneumovirus*, and *adenovirus*. In terms of bacterial detection, tNGS covered difficult-to-culture or unexpected species such as *Brucella melitensis*, *non-tuberculous mycobacteria*, and *Legionella pneumophila*. However, tNGS was less sensitive in detecting *Aspergillus* spp compared to the CMT. Further evaluation of the diagnostic performance of pathogens with a frequency count exceeding five times (*Mycobacterium tuberculosis*:4) showed higher diagnostic accuracy for *Pneumocystis jirovecii*, and *Legionella pneumophila*, but lower accuracy for *Aspergillus* spp, *Streptococcus pneumoniae*, and *rhinovirus* compared to CMT(**Table 3**). Additionally, in the diagnosis of *Epstein-Barr virus* (EBV), tNGS was able to detect more EBV in samples, resulting in significantly lower diagnostic accuracy than PCR. We conducted a comparative analysis of the RPM values between pathogens consistently detected by both methods and those detected exclusively by tNGS. The study findings indicate that the RPM values of *Pneumocystis jirovecii* detected exclusively by tNGS are comparatively lower, a phenomenon also observed in the analysis of viruses and bacteria (**Supplementary Figure 1**). This may be attributed to the higher sensitivity of the tNGS method.

**Table 3:** Diagnostic performance of tNGS in difference pathogens.

	tNGS					CMT					p-value
Pathogens	Sensitivity(95% CI)	Specificity (95%CI)	PPV (95% CI)	NPV (95%CI)	Accuracy (95%CI)	Sensitivity (95% CI)	Specificity (95%CI)	PPV (95% CI)	NPV (95%CI)	Accuracy (95%CI)	
***Pneumocystis jirovecii***	100.00% (82.41%-100.00%)	96.92% (92.36%-98.80%)	81.82% (61.48%-92.69%)	100.00% (97.04%-100.00%)	97.30% (93.26%-98.94%)	16.67% (5.84%-39.22%)	100.00% (97.13%-100.00%)	100.00% (43.85%-100.00%)	89.66% (83.63%-93.63%)	89.86% (83.95%-93.76%)	0.009
***Aspergillus* spp**	56.25% (33.18%-76.90%)	97.73% (93.53%-99.22%)	75.00% (46.77%-91.11%)	94.85% (89.76%-97.48%)	93.24% (88.01%-96.29%)	81.25% (56.99%-93.41%)	100.00% (97.17%-100.00%)	100.00% (77.19%-100.00%)	97.78% (93.67%-99.24%)	97.97% (94.21%-99.31%)	0.047
***Streptococcus pneumoniae***	83.33% (55.20%-95.30%)	78.68% (71.05%-84.72%)	25.64% (14.57%-41.08%)	98.17% (93.56%-99.50%)	79.05% (71.81%-84.83%)	83.33% (55.20%-95.30%)	98.53% (94.80%-99.60%)	83.33% (55.20%-95.30%)	98.53% (94.80%-99.60%)	97.30% (93.26%-98.94%)	<0.0001
***Klebsiella pneumoniae***	77.78% (45.26%-93.68%)	97.12% (92.83%-98.88%)	63.64% (35.38%-84.83%)	98.54% (94.83%-99.60%)	95.95% (91.44%-98.13%)	77.78% (45.26%-93.68%)	100.00% (97.31%-100.00%)	100.00% (64.57%-100.00%)	98.58% (94.98%-99.61%)	98.65% (95.21%-99.63%)	0.15
***Haemophilus influenzae***	100.00% (70.09%-100.00%)	93.53% (88.15%-96.56%)	50.00% (29.03%-70.97%)	100.00% (97.13%-100.00%)	93.92% (88.85%-96.77%)	44.44% (18.88%-73.33%)	100.00% (97.31%-100.00%)	100.00% (51.01%-100.00%)	96.53% (92.13%-98.51%)	96.62% (92.34%-98.55%)	0.27
***Staphylococcus aureus***	100.00% (60.97%-100.00%)	100.00% (97.37%-100.00%)	100.00% (60.97%-100.00%)	100.00% (97.37%-100.00%)	100.00% (97.47%-100.00%)	83.33% (43.65%-96.99%)	100.00% (97.37%-100.00%)	100.00% (56.55%-100.00%)	99.30% (96.15%-99.88%)	99.32% (96.27%-99.88%)	0.32
***Pseudomonas aeruginosa***	66.67% (30.00%-90.32%)	100.00% (97.37%-100.00%)	100.00% (51.01%-100.00%)	98.61% (95.08%-99.62%)	98.65% (95.21%-99.63%)	66.67% (30.00%-90.32%)	100.00% (97.37%-100.00%)	100.00% (51.01%-100.00%)	98.61% (95.08%-99.62%)	98.65% (95.21%-99.63%)	1.00
***Mycobacterium tuberculosis complex***	100.00% (51.01%-100.00%)	97.92% (94.05%-99.29%)	57.14% (25.05%-84.18%)	100.00% (97.35%-100.00%)	97.97% (94.21%-99.31%)	25.00% (4.56%-69.94%)	100.00% (97.40%-100.00%)	100.00% (20.65%-100.00%)	97.96% (94.17%-99.30%)	97.97% (94.21%-99.31%)	1.00
***Legionella pneumophila***	100.00% (51.01%-100.00%)	100.00% (97.40%-100.00%)	100.00% (51.01%-100.00%)	100.00% (97.40%-100.00%)	100.00% (97.47%-100.00%)	0.00% (0.00%-48.99%)	100.00% (97.40%-100.00%)	NA	97.30% (93.26%-98.94%)	97.30% (93.26%-98.94%)	0.044
**SARS-cov-2**	90.48% (71.09%-97.35%)	94.49% (89.06%-97.30%)	73.08% (53.92%-86.30%)	98.36% (94.22%-99.55%)	93.92% (88.85%-96.77%)	76.19% (54.91%-89.37%)	100.00% (97.06%-100.00%)	100.00% (80.64%-100.00%)	96.21% (91.44%-98.37%)	96.62% (92.34%-98.55%)	0.27
***Epstein-Barr Virus***	100.00% (67.56%-100.00%)	65.00% (56.79%-72.40%)	14.04% (7.29%-25.32%)	100.00% (95.95%-100.00%)	66.89% (58.97%-73.96%)	100.00% (67.56%-100.00%)	100.00% (97.33%-100.00%)	100.00% (67.56%-100.00%)	100.00% (97.33%-100.00%)	100.00% (97.47%-100.00%)	<0.0001
***Rhinovirus***	60.00% (23.07%-88.24%)	95.10% (90.24%-97.61%)	30.00% (10.78%-60.32%)	98.55% (94.87%-99.60%)	93.92% (88.85%-96.77%)	80.00% (37.55%-96.38%)	100.00% (97.38%-100.00%)	100.00% (51.01%-100.00%)	99.31% (96.17%-99.88%)	99.32% (96.27%-99.88%)	0.01
***Influenza A virus***	100.00% (56.55%-100.00%)	97.90% (94.01%-99.28%)	62.50% (30.57%-86.32%)	100.00% (97.33%-100.00%)	97.97% (94.21%-99.31%)	100.00% (56.55%-100.00%)	100.00% (97.38%-100.00%)	100.00% (56.55%-100.00%)	100.00% (97.38%-100.00%)	100.00% (97.47%-100.00%)	0.082

tNGS, targeted Next-generation sequencing; CMT, conventional microbiological tests; PPV, positive predictive value; NPV, negative predictive value; NA, not available. The difference of diagnostic accuracy based on the t-test of tNGS and CMT was marked as p-value. The names of the pathogens have been bolded.

### 3.4 Clinical diagnostic performance in different types of cancer

The performance of the tNGS method in the etiological diagnosis of pneumonia in different cancer patients was further explored. In patients with lung cancer, tNGS showed 80.68% of sensitivity and 93.33% of specificity (**Table 4**). In patients with gastrointestinal cancer and hematological malignancies, tNGS demonstrates a sensitivity exceeding 90% of tNGS. Whether in patients with lung cancer, gastrointestinal cancer, or hematological malignancies, the diagnostic accuracy of tNGS was higher than CMT. Compared with the overall patients, there was no significant difference in the diagnostic accuracy of tNGS among different cancer types (p>0.05). The distribution of pathogens among different cancer types was observed (**Figure 2C**). Most of the pathogens were identified in patients with lung cancer, accounting for about 60%, with 73.42% being bacterial pathogens. Patients with hematological malignancies accounted for 25% of identified pathogens, but only 11.39% were bacterial. In gastrointestinal cancer cases, the proportion of identified pathogens was 10.12%, with fungi being relatively rare at only 5.41%. *Pneumocystis jirovecii* and *Aspergillus* spp were more commonly found in patients with lung cancer and hematological malignancies, while common pathogens such as *Pseudomonas aeruginosa*, *Mycoplasma pneumoniae*, *Streptococcus pneumoniae*, and others were more prevalent in patients with lung cancer (**Figure 2D**). No differences were observed in the presence of *Klebsiella pneumoniae*, *Acinetobacter baumannii*, and *Mycobacterium tuberculosis* in patients with different types of cancer. Interestingly, a higher number of *severe acute respiratory syndrome coronavirus 2* infections were found in patients with hematological malignancies, but no differences were observed in the presence of other viruses among patients with different types of cancer.

**Table 4:** The diagnostic performance of tNGS in patients with difference type of cancer.

	Methods	Sensitivity(95% CI)	Specificity(95%CI)	PPV(95% CI)	NPV(95%CI)	Accuracy(95%CI)	p-value
**lung cancer**	**CMT**	55.68% (45.28%-65.61%)	100.00% (79.61%-100.00%)	100.00% (92.73%-100.00%)	27.78% (17.62%-40.89%)	62.14% (52.49%-70.91%)	0.0011
	**tNGS**	80.68% (71.22%-87.57%)	93.33% (70.18%-98.81%)	98.61% (92.54%-99.75%)	45.16% (29.16%-62.23%)	82.52% (74.06%-88.65%)	
**gastrointestinal cancer**	**CMT**	36.36% (15.17%-64.62%)	100.00% (34.24%-100.00%)	100.00% (51.01%-100.00%)	22.22% (6.32%-54.74%)	46.15% (23.21%-70.86%)	0.011
	**tNGS**	90.91% (62.26%-98.38%)	100.00% (34.24%-100.00%)	100.00% (72.25%-100.00%)	66.67% (20.77%-93.85%)	92.31% (66.69%-98.63%)	
**hematological malignancies**	**CMT**	70.37% (51.52%-84.15%)	NA	100.00% (83.18%-100.00%)	0.00% (0.00%-32.44%)	70.37% (51.52%-84.15%)	0.011
	**tNGS**	96.30% (81.72%-99.34%)	NA	100.00% (87.13%-100.00%)	0.00% (0.00%-79.35%)	96.30% (81.72%-99.34%)	

tNGS, targeted Next-generation sequencing; CMT, conventional microbiological tests; PPV, positive predictive value; NPV, negative predictive value; NA, not available. The difference of diagnostic accuracy based on the t-test of tNGS and CMT was marked as p-value. Bold formatting is applied to emphasize the subgroups and detection methods.

### 3.5 Clinical impact of tNGS: case report

A retrospective review of the treatment history of patients was conducted to further analyze the impact of tNGS on clinical treatment. It was found that tNGS results directly influenced antibiotic treatment decisions in many patients.

Case 1: A patient with lung cancer presented with symptoms of coughing and sputum production and was diagnosed with pneumonia at our hospital. The patient was treated with anti-infective agents, but no improvement was observed. A tNGS test for respiratory pathogens was performed, revealing the presence of *Rhinovirus C* and *Pneumocystis jirovecii*. Given the patient’s deteriorating pulmonary condition and the tNGS results, it was clinically considered that the infection was caused by both viral and fungal pathogens. Consequently, the patient was administered Levofloxacin and Piperacillin for anti-infective treatment, along with a combination of Sulfamethoxazole and Trimethoprim tablets for antifungal treatment, the patient showed signs of improvement.

Case 2: A patient with follicular lymphoma presented with fever and subsequently underwent a CT scan, which indicated symptoms of pulmonary infection. A tNGS test for respiratory pathogens detected *Pneumocystis jirovecii*. Symptomatic treatment with Sulfamethoxazole and Trimethoprim was administered. The patient has not experienced further fever and has shown symptomatic improvement following continued anti-infective treatment.

Case 3: A patient, post-debulking surgery for a malignant ovarian cancer, presented with symptoms of coughing and sputum production. Upon admission, biochemical tests and CT sacn indicated a pulmonary infection. tNGS test identified a specific pathogen: *Mycobacterium tuberculosis complex*. The patient was commenced on anti-tuberculosis therapy, which led to symptomatic relief. The patient was subsequently discharged following improvement.

These cases emphasize the important role of tNGS in guiding the diagnosis of infections in cancer patients and targeted antibiotic therapy, ultimately improving patient prognosis.

## 4 Discussion

Diagnosing pneumonia in cancer patients undergoing treatment is crucial, as it aids in managing patient infections and in decision-making regarding the timing of anti-cancer treatments. Targeted NGS is a common used for cancer-related gene detection, but its use for detecting infectious pathogens is a novel technique (Xia et al., 2023; Zhang et al., 2024). We conducted a retrospective analysis of the performance of this method in cancer patients. This study demonstrated that tNGS provides significantly higher sensitivity and a broader range of pathogen detection compared to CMT in cancer patients with pneumonia. Our findings highlight the potential of tNGS as a reliable diagnostic tool for managing infections in these patients.

As a new technology, understanding the clinical performance of tNGS is crucial. Previous studies have systematically evaluated the diagnostic value of tNGS in lower respiratory tract infections and pneumonia (Gaston et al., 2022; Zhang et al., 2024). However, research in immunocompromised patients has been relatively limited. Our study confirmed that tNGS is a more sensitive technique for diagnosing pneumonia in cancer patients compared to CMT methods (84.62% vs 56.92%, p<0.0001). tNGS also showed higher sensitivity in detecting bacteria, viruses, and fungi (Deng et al., 2023). These findings are consistent with previous studies, which indicated that the tNGS method maintains a high sensitivity in cancer patients with pneumonia (Deng et al., 2023; Mansoor et al., 2023). However, the increased sensitivity of tNGS may also lead to the detection of non-pathogenic microorganisms, resulting in reduced specificity (Rodino and Simner, 2024). This emphasized the importance of carefully interpreting tNGS results, as the study suggests (Fourgeaud et al., 2024; Yin et al., 2024), by incorporating the clinical context into the interpretation of tNGS results to avoid overdiagnosing non-pathogenic microorganisms. In particular, the broader detection of *human herpesvirus* in viral analysis had also led to a decrease in the accuracy of viral infection diagnosis. Although this might be related to the higher reactivation of *herpesviruses* in immunocompromised populations and the neglected of herpesvirus diagnosis in clinical practice, the detection of herpesviruses had not played a significant role in antibiotic decision-making (Huang et al., 2023; Jiang et al., 2023). Therefore, the ability of tNGS to detect a broader range of pathogens makes it a sensitivity diagnostic tool for cancer patients who are at risk of opportunistic infections.

In terms of pathogen detection performance, tNGS identified a significantly greater number of pathogens, whether in bacteria, fungi, or viruses. This was mainly due to the ultrasensitivity of the NGS method (**Supplementary Figure 1**), which was reflected in the detection of most pathogens (Deng et al., 2020; Li et al., 2021; Peng et al., 2021; Sun et al., 2021). Additionally, tNGS was able to detect some difficult-to-culture or unexpected pathogens, such as *Pneumocystis jirovecii*, *Legionella pneumophila*, and *Brucella melitensis*, which fully demonstrated the advantage of the NGS method’s broad coverage (**Supplementary Table 1**) (Zhang et al., 2024). However, it should be noted that tNGS was not as sensitive as the CMT method for *Aspergillus* spp, consistent with previous findings (Peng et al., 2021). Insufficient extraction of molds may be due to the simultaneous extraction of DNA and RNA required by tNGS. Moreover, CMT may have detected more using the galactomannan (GM) method, which might have resulted in insufficient detection when the *Aspergillus* spp load was low (Peng et al., 2021). Additionally, it was observed that the diagnostic accuracy of most pathogens was not much different from CMT, with better diagnostic accuracy for *Pneumocystis jirovecii* and *Legionella pneumophila* in tNGS. This may be due to the clinical application of PCR methods that covered these pathogens, making their detection limits comparable to tNGS (Murphy et al., 2020). In common pathogens such as *Klebsiella pneumoniae* and *Pseudomonas aeruginosa*, the performance of both methods was similar, whis is also related to the widespread use of multiplex PCR (Zhang et al., 2022). In summary, tNGS was a more sensitive and broad-spectrum detection method compared to CMT, and except for *Aspergillus* spp, this method could provide more evidence to support clinical diagnosis.

Several studies have discussed the impact of mNGS on etiological diagnosis and antibiotic adjustment. Sun and colleagues reported that mNGS can guide antibiotic adjustments in critically ill pneumonia patients, with 87% of immunocompromised patients undergoing antibiotic adjustments based on mNGS results (Sun et al., 2021). Additionally, Wei et al.’s study mentioned that tNGS played a positive role in the pathogen diagnosis of 72.7% of patients and may have led to antibiotic treatment adjustments in 17% of patients (Wei et al., 2024). Our study also confirmed these findings. Given that the tNGS method costs only about half as much as mNGS and is even comparable to the price of multiplex PCR, the application of tNGS in primary or secondary testing may significantly enhance diagnostic accuracy and reduce the misuse of antibiotics. Our study also explored the diagnostic performance of tNGS in different cancer types, with no significant differences observed. This is similar to the impact of antibiotics on NGS detection performance, where nucleic acid levels are less influenced by patient characteristics (Azoulay et al., 2020). However, when comparing the distribution of pathogens among different cancer types, we found that fungi were detected more frequently in patients with lung cancer and hematological malignancies, which may be related to the higher proportion of these populations. Additionally, we observed that *Streptococcus pneumoniae*, *Haemophilus influenzae*, *Staphylococcus aureus*, and *Pseudomonas aeruginosa* occurred more frequently in lung cancer patients compared to those with hematological malignancies or gastrointestinal cancer. These pathogens are more likely to colonize the respiratory tract and may be more prone to cause infections due to the immune impairment caused by lung cancer (Plummer et al., 2016; van Elsland and Neefjes, 2018; Hatta et al., 2021). Of course, this may also be related to the uneven distribution of patients with different cancer types.

This study has several limitations. Firstly, due to the retrospective nature of this study and the relatively small sample size, potential biases may exist, such as the diagnostic value for pathogens with lower incidence rates, like Mycoplasma pneumoniae and the Mycobacterium tuberculosis complex, which require a larger population to clarify … Secondly, the PCR detection of DNA viruses, especially *herpesviruses*, was determined by clinical needs, which may introduce bias in the evaluation of the performance of DNA virus detection. Lastly, the distribution of patients with different types of cancer in our cohort was relatively concentrated, suggesting that the conclusions regarding the pathogen preferences of different cancer types need to be verified in a larger cohort.

## 5 Conclusion

In conclusion, tNGS shows great promise as a diagnostic tool for detecting infections in cancer patients. It offers higher sensitivity and broader pathogen detection capabilities compared to conventional methods. However, despite its limitations, tNGS can significantly enhance the management of infections in immunocompromised patients, potentially leading to better clinical outcomes. Future research should focus on optimizing the specificity of tNGS and integrating it into routine clinical practice to fully leverage its diagnostic potential.

## Data Availability

The data presented in the study are deposited in the China National Center for Bioinformation - National Genomics Data Center repository, accession number PRJCA035966.

## Supplementary material

Supplementary Figure 1 Further analysis to additional detections by RPM of tNGS. The results were grouped based on whether the CMT was positive or negative (labeled as CMT+/tNGS+ or CMT-/tNGS+). *: p<0.05, **: p<0.01, ***: p<0.001.
