# Supplementary figures and images for: Clinical application of targeted next-generation sequencing in pneumonia diagnosis among cancer patients
Source: Front Cell Infect Microbiol. 2025 Feb 18;15:1497198. doi: 10.3389/fcimb.2025.1497198 (PMC11876428; doi:10.3389/fcimb.2025.1497198)

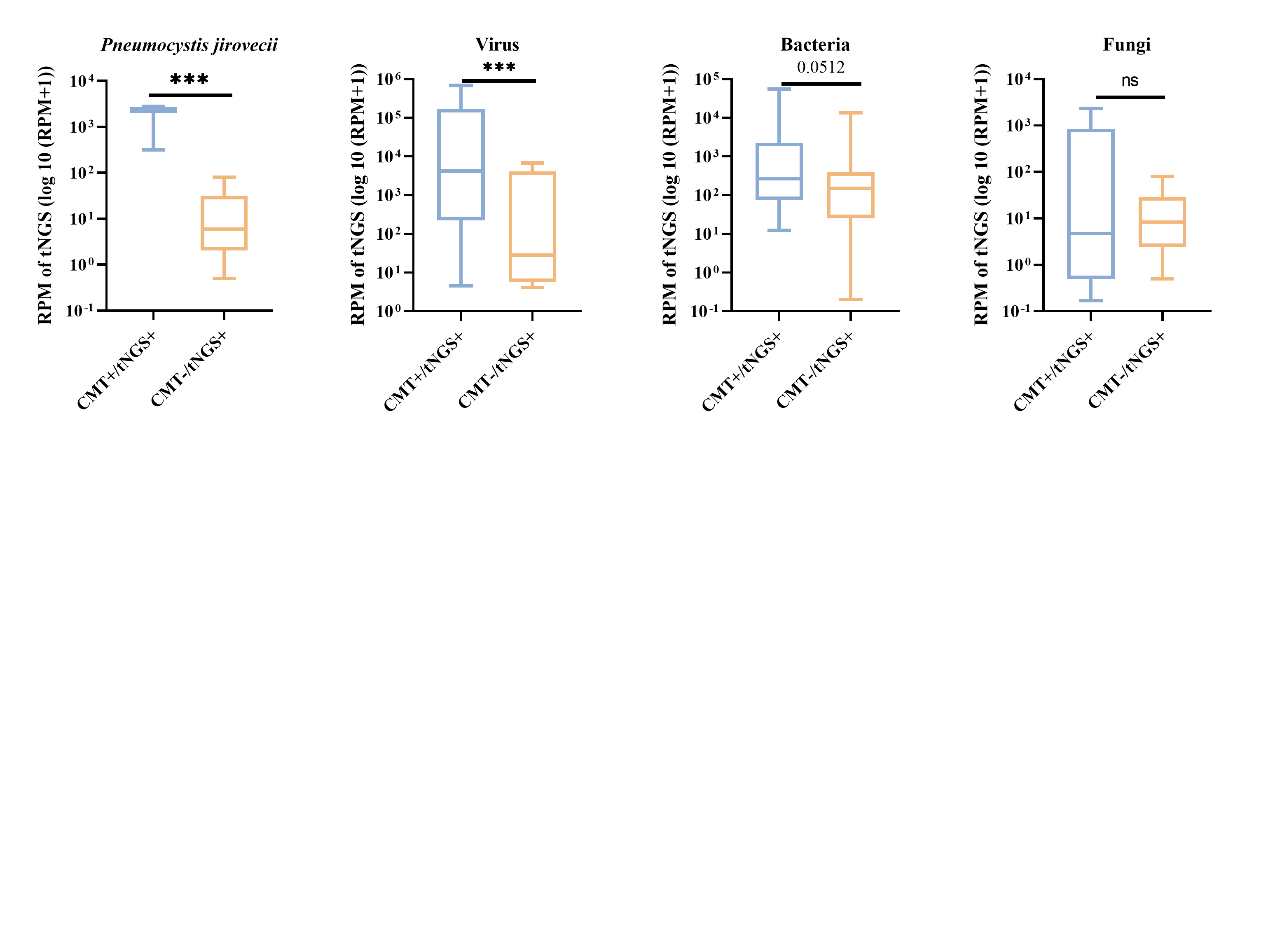

Supplement: Supplementary Figure 1 — Further analysis to additional detections by RPM of tNGS. The results were grouped based on whether the CMT was positive or negative (labeled as CMT+/tNGS+ or CMT-/tNGS+). *: p<0.05, **: p<0.01, ***: p<0.001. [file Image1.tif]
